# Pre and post chemotherapy evaluation of breast cancer patients: Biochemical approach of serum selenium and antioxidant enzymes

**DOI:** 10.22088/cjim.11.4.403

**Published:** 2020

**Authors:** Fatemeh Pakmanesh, Daryoush Moslemi, Soleiman Mahjoub

**Affiliations:** 1 Student Research Committee, Babol University of Medical Sciences, Babol, Iran; 2 Cancer Research Center, Health Research Institute, University of Medical Sciences, Babol, Iran; 3 Cellular and Molecular Biology Research Center, Health Research Institute, Babol University of Medical Sciences, Babol, Iran; 4 Department of Clinical Biochemistry, School of Medicine, Babol University of Medical Sciences, Babol, Iran

**Keywords:** Breast cancer, Chemotherapy, Catalase, Glutathione Peroxidase, Glutathione Reductase, Superoxide Dismutase, Selenium

## Abstract

**Background:**

Chemotherapy for treatment of breast cancer uses some drugs to target and destroy the cancer cells. However, most of antineoplastic treatments are non-specific and the innate cells will be damaged. In this study, the effect of adriamycin/cytoxan (AC) chemotherapy on the status of antioxidant enzymes and Se levels in breast cancer patients was evaluated.

**Methods:**

A prospective study, includes 50 breast cancer patients treated with AC chemotherapy (adriamycin 60 mg/m^2^, cytoxan 600 mg/m^2^) from July 2016 until March 2017. First sampling was obtained before chemotherapy and the second, after 3 cycles of the intervention. Antioxidant enzyme activities (catalase, glutathione peroxidase, glutathione reductase and superoxide dismutase) and selenium (Se) levels in serum were measured by spectrophotometry and atomic absorption methods, respectively. Age, BMI, familial history, stage and grade of cancer, tumor site, type of surgery, estrogen, progesterone and HER2 receptors, were recorded from each patient. Paired-t test was employed for comparing the data before and after chemotherapy. Age and disease stages were compared by independent t-test.

**Results:**

After 3 courses of chemotherapy, a significant decrease was observed in antioxidant enzymes and also Se (p<0.001). These studied indices were not significant in different age groups (≤48, >48) and stages of disease (early, advanced).

**Conclusion:**

Our findings show that the AC chemotherapy in the breast cancer patients result in drastic changes in oxidant/antioxidant system of the body, especially reduction of Se levels and antioxidant enzymes activities. However, it seems that these changes are not necessarily dependent on the age and disease stage.

Breast cancer has the highest prevalence among the women all around the world and has been the most common malignancy among Iranian women in recent decades (1, 2). Currently, half of breast cancer cases and about 60% of its mortalities occurs in the developing countries (3). In Iran, one out of 10-15 women are suffering from breast cancer (4). Among all the effective factors of growth and metastasis of tumor, oxidative stress, decrease of antioxidant enzymes and deficiency of some trace elements such as selenium (Se) can play an important role in cancer onset by damaging DNA and interfering with inter-cellular pathways (5). Free radicals and reactive oxygen species (ROS) are accounted as cellular damage factors due to oxidative stress which can result in lipids, cell membrane, proteins and genomic content deformation as the carcinogen (6). 

Se is one of the most essential trace elements due to its important roles in prevention of cancer, antioxidant activities and immune system enhancement. It is proposed that the administration of Se (100-200 μg/Day) can prevent genetic damages and cancer progress in human (7). During chemotherapy, various anti-cancer drugs provoke antioxidant system of the body including enzymic and non-enzymic antioxidants; leading to alterations in biological function of the cell. 

Chemotherapy regimens can decrease the antioxidants in the body. However, some drug combinations can promote the antioxidant status (8).

The study of Atukeren et al. in 2010 included 30 patients (before treatment and after first and second chemotherapy course) and 20 healthy cases. Their results showed the reduction of antioxidant enzyme activities, glutathione peroxidase (GP_x_) and glutathione reductase (GR) in patients as compared with control group and the same results were observed after chemotherapy (9). Kasapovic et al. conducted chemotherapy with 5-fluorouracil, adriamycin and cytoxan (FAC) based on superoxide dismutase (SOD), GP_x_ and GR levels in 58 cases of breast cancer and 60 healthy cases. Their studies showed that antioxidant enzyme activities decreased in response to the FAC chemotherapy. Overall, FAC chemotherapy and radiotherapy increase the oxidative shift (10). The decreased level of Se in breast cancer patients compared with pretreatment was reported as the effect of radiotherapy (11). 

Bindary et al. investigated the effect of chemotherapy on catalase (CAT) and SOD enzymes in breast cancer patients. They concluded that chemotherapy reduces CAT and SOD enzyme levels (12). Ragab et al. conducted a research in malignant and benign breast cancer patients. Their study revealed a significant increase in the concentration of trace elements such as Pb, Ni, Fe, Cr and Cd in malignant tissues of the patients in comparison with control group (p<0.001). A significant decrease was also observed in CAT and GR in patients as compared with the control group (13).

As we have known, any reported data about Se and antioxidant enzyme variations in treatment courses (after 3 cycles) have not been reported previously. Regarding the ambiguities about the effect of chemotherapy drugs on the antioxidant system of the body, this study aimed to investigate the antioxidant indices before and after 3 courses of the AC chemotherapy based on the patients’ age and the stages of the disease.

## Methods

**Study Population and Sample Collection: **This cross-sectional study was conducted in 50 breast cancer patients, age 31-74 years, referred to *Shahid *Rajayee* Hospital, Babolsar, North of Iran, between *July 2016 until March 2017*. *The study was approved by the Ethics Committee of Babol University of Medical Sciences (MUBABOL, HRI, REC.1395.21) and the written informed consent was obtained from all the participants. The patients were exposed to chemotherapy by the AC protocol (adriamycin 60 mg/m^2^, cytoxan 600 mg/m^2^), intravenously once every 3 weeks. Participants with smoking, coffee and alcohol consumption, those who used vitamins or Se and other antioxidants (as supplements), contraceptive pills, and those who suffered from diabetes mellitus, thalassemia, inflammatory disease and other malignant diseases were excluded from the study. Every patient has undergone two blood sampling. First sampling performed 4 weeks after surgery (before the initiation of chemotherapy) and second, 3 courses after chemotherapy (usually, after 9 weeks from first chemotherapy). Peripheral blood sampling performed and after serum separation, the samples were immediately transported to the Biochemistry Research Laboratory and stored at−80^o^C for biochemical tests.

**Measurement of SOD Activity: **Serum SOD activity in the specimens (U/mL) was measured using ZellBio GmbH assay kit (Ulm, Germany) based on colorimetric method according to manufacturer's protocol. The absorbance of samples was determined at time 0 and 2 mins using microplate reader (Stat Fax, Awareness, USA) at 420 nm. 

**Measurement of GR & GPx Activities: **Activities of GR and GPx measurement were done based on colorimetric assay with microplate reader (Stat Fax, Awareness, USA) using ZellBio GmbH assay kits (Ulm, Germany), according to the manufacturer’s protocol.

**Measurement of** **CAT** **Activity: **In this study, CAT enzyme assay was assessed according to the Aebi method (14). Briefly, 1𝜇L of sample was added to 1 ml of working reagent (333 𝜇L of 30 mmol H_2_O_2_ and 666 𝜇L of 50 mM phosphate buffer saline, pH 7). The analysis was performed using UV Quartz cuvette. Once adding the substrate, reaction started and after 1 minute the absorbance was read at 240 nm in room temperature (25ºC). Catalase activity was determined according to volume of H_2_O_2_ consumption per minute at room temperature (25 ºC).

**Determination of Selenium by GFAAS: **Concentration of Se was determined by graphite furnace atomic absorption spectroscopy (GFAAS). Analytical parameters used for this procedure were: 196 nm wavelength, 0.4 nm bandwidth and 5 mA lamp current. Detection limit was 1.5 ppb and working range 3.15-100 ppb. To produce standard serial dilutions, a first standard stock of SeO_2_ (Merck, 1000 ppm) was prepared by dissolving 0.07 gr SeO_2_ in 0.1% nitric oxide up to 50 ml. The standard serial dilutions were prepared using stock solution as (3.12, 6.25, 12.5, 25, 50 and 100 ppb). The samples were diluted in 1.2 using 0.1% nitric acid. The prepared standards and samples were injected into the apparatus and according to the standard curve, the concentration of the samples was calculated in terms of ppb.

## Results

Clinicopathological data of 50 breast cancer patients (ages 48±17), undergoing the AC chemotherapy, are presented in table 1. Information of table 1 are related to pre-chemotherapy stage which are analyzed statistically. With regard to the files of these patients and performance of precise statistical analysis, frequencies of indices such as hormonal receptors (Her-2, ER and PR), lymph node involvement (N), tumor size (T) and metastasis (M) along with patient frequency based on their grade and stage were investigated. The impact of chemotherapy on SOD, CAT, GP_x_ and GR enzymes and Se trace element are listed in table 2. The indices of this table are related to measurements before and after chemotherapy.

Based on table 2, a significant decrease was observed in measured antioxidant enzymes such as CAT, GP_x_, GR and SOD. After chemotherapy, mean GR levels were 31 U/L lower than before (p<0.001). A similar decrease was observed in mean GPx, CAT and SOD levels after chemotherapy compared with before (11.6 U/ml, 2.7 U/ml and 6.7 U/ml respective). A significant decrease was also observed in mean Se level (2.2 µg/L, p<0.001). After chemotherapy, no significant variation was observed in BMI index. Based on the disease stage, the effect of chemotherapy on mean and standard deviation of antioxidant enzymatic indices as well as Se levels are presented in table 3.

According to the disease stage, after chemotherapy, also there was a significant reduction in antioxidant enzymes as well as Se levels, however, there were no significant differences in the basic levels in patients (p >0.05), except the GPx levels in patients before chemotherapy which was lower in the advance stage , compared with the early stage. In table 4,the mean and standard deviation of antioxidant enzymatic indices and selenium before and after chemotherapy were compared. Studied indices showed no significant variation in different ages (≤48 and>48) and stages (early, advanced) groups as mentioned in tables 3 and 4.

**Table 1:** Clinicopathological data of breast cancer patients

Characteristics	N	%
**Number of patients**	50	-
**Age (median)**	48	-
**Rang age**	31-74	-
Cancer site **Right breast** **Left breast**	23 27	46 54
Family background **Present** **Absent**	9 41	18 82
Surgery **Mastectomy** **Lumpectomy**	41 9	82 18
ER status **Negative** **Positive**	16 34	32 68
PR status **Negative** **Positive**	17 33	34 66
HER-2 status **Negative** **Positive**	32 18	64 36
Lymph node involvement **N**_0_ **N**_1_ **N**_2_ **N**_3_	14 23 7 6	28 46 14 12
Tumor size **T**_1_ **T**_2_ **T**_3_ **T**_4_	7 29 13 1	14 58 26 2
Metastasis **M**_0_ **M**_1_ **M**_X_	34 8 8	68 16 16
Grade **G**_1_ **G**_2_ **G**_3_	12 35 3	24 70 6
Stage **Early** **Advanced**	32 18	64 36

ER, Estrogen Receptor; PR, Progesterone Receptor; Her-2, Human epidermal receptore-2; Early stage, stage I, II; Advanced stage, stage III, IV.

**Table 2:** Mean and standard deviation of antioxidant enzymatic indices and selenium before and after chemotherapy

	Mean±SD		P-value
	Before chemotherapy	After chemotherapy	
**GR, U/L**	82.8±36.7	51.0±23.6	<0.001
**GP**_X_ **, U/mL**	32.8±11.83	21.2±7.72	<0.001
**CAT, U/mL**	19.7±2.4	17.0±2.1	<0.001
**SOD, U/mL**	24.2±4	17.5±3.7	<0.001
**Se, µg/L**	8.8±2.4	6.6±1.4	<0.001
**BMI, Kg/m** ^2^	27.7±3.8	27.8±3.7	0.48

GR, Glutathione Reductase; GP_X_, Glutathione peroxidase; CAT, Catalase; SOD, Superoxide Dismutase; Se, Selenium; BMI, body mass index.

**Table 3:** Mean and standard deviation of antioxidant enzymatic indices and selenium before and after chemotherapy based on the disease stage

	Groups	Early stage *(*32=n*)*	Advanced stage *(*18=n*)*	P-value
**GR, U/L**	Before	81.1±37.2	85.8±36.6	0.669
	After	46.3±22.8	59.2±23.3	0.064
	p-value	<0.001	0.019	
**GP**_X_ **, U/mL**	Before	36.0±12.5	27.0±7.8	0.009
	After	22.4±8.6	19.0±5.3	0.133
	p-value	<0.001	<0.001	
**CAT, U/mL**	Before	19.8±2.6	19.7±2.1	0.928
	After	17.2±2.2	16.7±1.9	0.374
	p-value	<0.001	<0.001	
**SOD, U/mL**	Before	23.8±3.8	25.0±4.4	0.338
	After	17.9±3.9	16.6±3.3	0.347
	p-value	<0.001	<0.001	
**Se, µg/L**	Before	8.7±1.7	9.1±3.3	0.529
	After	6.8±1.4	6.3±1.3	0.247
	p-value	<0.001	<0.001	
**BMI, Kg/m** ^2^	Before	27.0±3.6	29.0±3.8	0.075
	After	27.1±3.7	29.1±3.6	0.074
	p-value	0.456	0.818	

GR, Glutathione Reductase; GP_X_, Glutathione peroxidase; CAT, Catalase; SOD, Superoxide Dismutase; Se, Selenium; BMI, body mass index.

**Table 4:** Mean and standard deviation of antioxidant enzymatic indices and selenium before and after chemotherapy in two age groups

	Groups	Age groups ≤48 *(*(n=28	Age groups >48 *(*n=22*)*	P-value
**GR, U/L**	Before	81.7±34.6	84.2±40	0.811
	After	48.5±21.3	54.1±26.3	0.406
	p-value	<0.001	0.009	
**GP**_X_ **, U/mL**	Before	29.9±10.4	36.4±12.7	0.055
	After	19.9±7.2	22.8±8.2	0.194
	p-value	<0.001	<0.001	
**CAT, U/mL**	Before	19.6±2.4	19.9±2.4	0.654
	After	16.7±2.3	17.4±1.9	0.235
	p-value	<0.001	<0.001	
**SOD, U/mL**	Before	25.3±3.7	22.9±4.1	0.042
	After	17.4±3.6	17.6±4	0.877
	p-value	<0.001	<0.001	
**Se, µg/L**	Before	8.3±2.4	9.5±2.2	0.085
	After	6.3±1.3	7.1±1.5	0.055
	p-value	<0.001	<0.001	
**BMI, Kg/m** ^2^	Before	28.2±3.8	27.1±3.8	0.312
	After	28.3±3.7	27.2±3.8	0.298
	p-value	0.581	0.680	

GR, Glutathione Reductase; GP_X_, Glutathione peroxidase; CAT, Catalase; SOD, Superoxide Dismutase; Se, Selenium; BMI, body mass index.

## Discussion

Chemotherapy drugs can induce the different variations in body cells and metabolisms which can result increase in oxidative stress and decrease in antioxidant capacity. Our findings demonstrated antioxidant enzymes such as SOD, CAT, GP_x_ and GR along with Se element that significantly decreased in the serum of the patients after 3 courses of the AC chemotherapy.

It is clear that the increase of oxidative stress in breast cancer is correlated with disease severity. Oxidative stress in these patients may discharge the antioxidant capacity. Therefore, it seems that the antioxidant status is a determining factor in tumors’ sensitivity toward oxidative stress during treatment with anticancer medications (15). The study of Aturkeren et al. on 30 patients before treatment and after first and second chemotherapy courses using adjuvant anthracycline-based chemotherapy, showed significant decrease in activities of antioxidant enzymes such as GP_x _and GR (9). In our study, GP_x_ and GR showed significant decrease after the AC chemotherapy. Our investigation was after the third chemotherapy course and we also examined the serum levels of Se as trace element. In the study of Ragab et al. in 2014, a significant decrease was reported in CAT and GR levels (13). Similar results were also observed in our study. However, our study has the advantage of investigating the antioxidant indices and Se element before and after chemotherapy. In the research of Kasapovic et al. in 2010 revealed the reduction of antioxidant enzyme activities especially SOD in breast cancer patients (10). Arjmandi et al. reported the decreased serum level of Se in breast cancer patients after radiotherapy as compared with before treatment (11). Franca et al. reported significant decrease in serum levels of Se in patients with breast cancer after treatment with external beam radiotherapy (16). They did not investigate other indices such as oxidant and antioxidant markers. 

In the present study, antioxidant enzymes and Se trace element were examined before and after the AC chemotherapy. In addition, we investigated the relationship between the antioxidant enzymatic markers and Se based on age, BMI and disease stage before and after AC chemotherapy. Our observations revealed the significant decrease of Se and antioxidant enzyme activities in the patients after chemotherapy. Other studies on oxidative stress in breast cancer patients have supported our finding (17). Our results showed that the activities of GR, CAT, SOD enzymes and Se element did not significantly change between the early and advanced stages of breast cancer. Although, the activity of GP_x_ in the advanced stages of the disease significantly decreased. Additionally, in both of the early and advanced stages of breast cancer, all antioxidant indices significantly decreased after 3 cycles of AC chemotherapy. The activities of GR, CAT, SOD enzymes and Se element did not significantly change in the two age groups (>48 and ≤48) of breast cancer. Yet, in both of the age groups of breast cancer, it significantly decreased in all antioxidant indices after AC chemotherapy. 

Se is an essential trace element which plays a crucial role in breast cancer prevention *via* its antioxidant effects (11, 18). Design of chemotherapy diets which is a combination of antioxidants with chemotherapy drugs should be closely investigated from the beginning of the treatment. Antioxidant complements can decrease the risk of tumor recurrence in the chemotherapy-treated women (9).

Another study reports that the simultaneous application of antioxidants and chemotherapy factors have potentials in weakening the chemotherapy or expression of the enzymes which can destroy the toxicity of cytotoxic factors (15). Nonetheless, further studies are needed to confirm this study.

Our previous study indicated that the AC chemotherapy increased the oxidative stress in breast cancer patients. We showed that higher stages of breast cancer are associated with significant increases of malondialdehyde as lipid peroxidation marker (19). The present study demonstrated the significant decrease of antioxidant enzymes and Se level in serum of the patients after 3 courses of the AC chemotherapy. Therefore, the present finding supports our previous study. The limitation of this study was the limited number of breast cancer patients treated by AC chemotherapy. The advantage of the present study is simultaneous investigation of all major antioxidant enzymes including SOD, CAT, GP_x_ and GR and also Se trace element. In addition, we measured and compared these indices in the early and advanced stages of breast cancer.

It seem that the present study is the first study that investigated Se and all major antioxidant enzymes after 3 courses of the AC chemotherapy simultaneously. We suggest that further studies be made to evaluate these indices after 6 courses of the AC-T (adriamycin 60 mg/m2, cytoxan 600 mg/m^2^, taxotere 80 mg/m^2^) chemotherapy and other regimens of chemotherapy with bigger sample size.

In conclusion the findings of the present study demonstrated the alteration in oxidant/antioxidant system of the body and drastic decrease of antioxidant ability after 3 courses of AC chemotherapy. Although it seems that these variations are not necessarily dependent on the age and disease stage; It is proposed that after the end of chemotherapy, the body antioxidant system should be reinforced by antioxidant compliments and enrichment of dietary natural antioxidant agents. It seems that by this way, the complications due to severe oxidative stress in other tissues and organs can be prevented.
